# Correction to “Translational Medicine in Alzheimer's Disease: The Journey of Donanemab From Discovery to Clinical Application”

**DOI:** 10.1002/cdt3.70063

**Published:** 2026-08-21

N. Jayaprakash and K. Elumalai, “Translational Medicine in Alzheimer's Disease: The Journey of Donanemab From Discovery to Clinical Application,” *Chronic Diseases and Translational Medicine* 11, no. 2 (2025): 105–116. https://doi.org/10.1002/cdt3.155.

In the published article, there were errors in the reference list. References 25 and 37 were duplicated. The correct reference should read:

25. S. Shcherbinin, C. D. Evans, M. Lu, et al., “Association of Amyloid Reduction After Donanemab Treatment With Tau Pathology and Clinical Outcomes: The TRAILBLAZER‐ALZ Randomized Clinical Trial,” *JAMA Neurology* 79, no. 10 (2022): 1015. https://doi.org/10.1001/jamaneurol.2022.2793.

Additionally, the bibliographic information for References 28 and 33 was incorrect. The correct references should read:

28. S. L. Lowe, C. Duggan Evans, S. Shcherbinin, et al., “Donanemab (LY3002813) Phase 1b Study in Alzheimer's Disease: Rapid and Sustained Reduction of Brain Amyloid Measured by Florbetapir F18 Imaging,” *Journal of Prevention of Alzheimer's Disease* 8, no. 4 (2021): 414–424. https://doi.org/10.14283/jpad.2021.56.

33. G. D. Rabinovici, D. J. Selkoe, S. E. Schindler, et al., “Donanemab: Appropriate Use Recommendations,” *Journal of Prevention of Alzheimer's Disease* 12, no. 5 (2025): 100150. https://doi.org/10.1016/j.tjpad.2025.100150.

The errors occurred during manuscript preparation and reference management. For Reference 28, the DOI was correct; however, a minor error in the article title was inadvertently retained from the imported Mendeley reference record and has now been corrected after verification with the published source. For Reference 33, the reference was inadvertently cited using information from the ACCP Abstract Booklet (Clinical Pharmacology in Drug Development) instead of the appropriate published article and has now been replaced with the correct citation. AI‐assisted reference‐generation tools were not used to generate these incorrect references.

These corrections are limited to the reference list and do not affect the scientific content, interpretation, or conclusions of the article.

The authors apologize for these errors.

